# A Rare Colon Polypectomy Diagnosis: Description of a Unique Investigational Journey and Literature Review of Burkitt Lymphoma

**DOI:** 10.7759/cureus.32293

**Published:** 2022-12-07

**Authors:** Gregory-Thomas C Stanger, Xiaolan Tang, Leora Frimer, Melanie N Rayan, Uma G Iyer

**Affiliations:** 1 Internal Medicine, University of Central Florida College of Medicine, Graduate Medical Education/HCA Florida North Florida Hospital, Gainesville, USA

**Keywords:** non-hodgkin lymphoma, starry-sky appearance, c-myc, b-cell lymphoma, sporadic burkitt lymphoma

## Abstract

The presentation of Burkitt lymphoma (BL) is variable and highly dependent on its subtype. It often presents with non-specific symptoms and may appear in the jaw, bone marrow, liver, spleen, kidneys, ovaries, and gastrointestinal tract. This case describes a 50-year-old male who presented with abdominal pain and was eventually found to have Burkitt lymphoma, diagnosed by biopsy of a cecal polyp. Herein, we explore the diagnostic journey to BL and review the literature regarding its unique manifestations and thus the diagnostic challenge it can present.

## Introduction

The clinical presentation of Burkitt lymphoma (BL) is non-specific, which can make its diagnosis challenging. It is crucial to make a histologic diagnosis to distinguish BL from other aggressive B-cell lymphomas. Patients with sporadic BL usually present with advanced-stage and bulky abdominal disease caused by a short doubling time of the tumor [1]. Here, we describe a 50-year-old patient who presented with right upper quadrant pain and weight loss, with eventual colonoscopy leading to a histologically-confirmed diagnosis of BL via a cecal polyp. This highlights the highly variable presentation of BL and that colonoscopy is one means by which diagnosis can be made.

## Case presentation

A 50-year-old, previously healthy, African American male presented to the emergency department for a two-week history of progressively worsening right upper quadrant (RUQ) abdominal pain. The pain was described as intermittent, without radiation, worsened by movement, coughing, and deep inspiration, and unchanged by food consumption. No relieving factors were identified. He had associated nausea and non-bilious non-bloody emesis every other day and a 20-pound weight loss over the prior two weeks which he attributed to decreased appetite and abdominal pain. He denied recent illnesses, prior surgeries, travel, chest pain, rashes, insect bites, Covid-19 exposure, diarrhea, constipation, hematochezia, or hematemesis. He denied a prior history of HIV or hepatitis and reported he had tested negative for HIV on discharge from an incarceration facility five years prior.

Initial physical examination was remarkable for sinus tachycardia of 140 beats per minute and mild right upper quadrant tenderness. An abdominal ultrasound (US) revealed a completely decompressed gallbladder. Computed tomography (CT) without contrast of the abdomen and pelvis revealed hepatosplenomegaly, minimally elevated common bile duct diameter of 6.5 mm, gallbladder wall thickening without evidence of cholelithiasis, and trace fluid along the inferior right hepatic lobe. His initial chemistry panel was pertinent for a serum creatinine of 1.3 mg/dL (0.55-1.30 mg/dL), gamma-glutamyl transpeptidase (GGT) of 435 IU/L (5-85 IU/L), alanine aminotransferase (ALT) 63 U/L (16-61 U/L), alkaline phosphatase (ALP) 341 IU/L (45-117 U/L), direct bilirubin 2.9 mg/dL (0.0-0.2 mg/dL), and total bilirubin of 3.6 mg/dL (0.2-1.0 mg/dL) and his complete blood count for a white count of 3.6 x 10^9^/L (4.0-10.5 x 10^9^/L), hemoglobin of 12.5 g/dL (13.7-17.5 g/dL), platelet count of 62,000 x 10^9^/L (150-400 x 10^9^/L), and increased atypical lymphocytes of 15%. His lactic acid was 2.9 mmol/L (0.5-2.2 mmol/L). He was given 30 cc/kg of IV fluids and ondansetron was ordered in the Emergency Department.

Repeat labs showed an acute kidney injury with a serum creatinine of 1.92 mg/dL. Magnetic resonance cholangiopancreatography (MRCP) revealed a small volume of free fluid in the pancreatic duodenal groove and surrounding liver with a slight collapse of the hepatic veins. Upon review of images with the radiologist, gastroenterology planned to perform a colonoscopy for non-specific cecal inflammation in the setting of RUQ abdominal pain, transaminitis, and hepatosplenomegaly. The following labs were unremarkable: Anti-nuclear antibody (ANA) and autoimmune markers, hepatitis A/B/C, Human Immunodeficiency Virus (HIV), Syphilis, COVID-19, Monoscreen, Serum Protein Electrophoresis (SPEP)/Immuno-Protein Electrophoresis (IPEP) with immunofixation, Hemochromatosis DNA, and urine drug screen. The patient underwent a colonoscopy on day 4 with the removal of a 10mm cecal polyp and an 18mm ascending polyp which was sent to pathology. With continuous intravenous fluids, his serum creatinine initially improved from 1.92 mg/dL to 1.7 mg/dL. Peripheral blood smear analysis revealed immature granulocytes and tear drop cells/target cells without blast cells, suggestive of liver disease and possible bone marrow dysfunction.

The patient’s pain persisted, prompting a repeat CT of the abdomen and pelvis, revealing persistent hepatosplenomegaly with nonspecific fluid in the paracolic gutter and pelvis. The repeat abdominal US showed hepatic enlargement with hypoechoic liver tissue and a dilated common bile duct up to 8mm. Liver physiology was suggestive of Budd-Chiari given the collapse of hepatic veins on MRCP without evidence of a blood clot seen on imaging. Given these findings, accompanied by worsening symptoms, and a coincidental total bilirubin elevation to 10 mg/dL on day 5, General Surgery was consulted. Due to his elevated Model for End-Stage Liver Disease (MELD) score of 26, a shunt was noted to be contraindicated.

**Figure 1:**
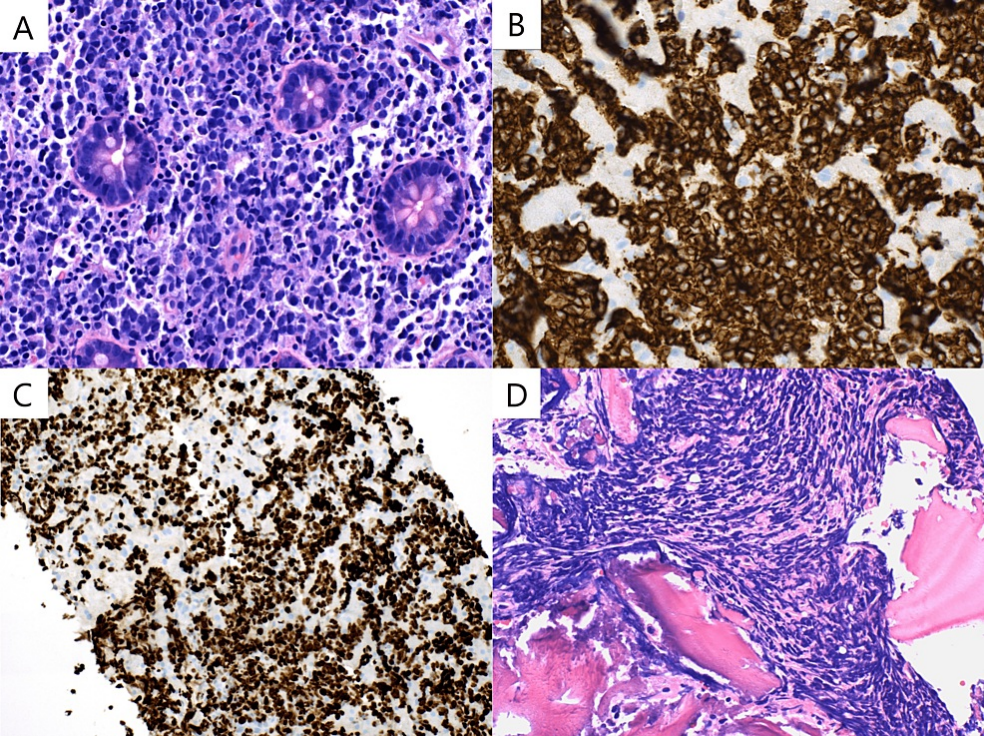
A) Hematoxylin & Eosin (H&E) stain of colonic cecal biopsy at 400X magnification: Intermediate-sized atypical lymphocytes infiltrating the lamina propria admixed with a few small lymphocytes. There is expansion within the crypts. Follicles of germinal centers are not seen; B) CD20 immuno-stain at 400X magnification: B-cell marker positive in the abnormal lymphocytes within the liver; C) KI-67 Proliferation Index in the liver at 200X magnification showing >90%; D) H&E stain at 400X magnification showing markedly hypercellular bone marrow with absent background trilineage hematopoiesis, involved by B-cell lymphoma.

Trans-jugular biopsy of the liver and bone marrow biopsy were performed on day 9. The cecal polyp biopsy revealed an aggressive B-cell lymphoma, germinal Cell B-cell subtype, with a high proliferation rate as observed in Figure 1A. FISH (Fluorescence in situ hybridization) studies and EBER (Epstein-Barr virus-encoded small RNAs) hybridization were ordered, later showing a positive MYC translocation with no IgH/BCL2 translocation and BCL6 rearrangement, consistent with Burkitt lymphoma.

Transjugular liver biopsy and bone marrow biopsy confirmed an aggressive B-Cell Lymphoma with blastoid morphology, consistent with Burkitt Lymphoma as shown in Figure 1B-1D. Before the final pathology reports returned, the patient's clinical status deteriorated with a serum creatinine of 3.81 mg/dL, total bilirubin of 24 mg/dL, and increased work of breathing. Acute intermittent hemodialysis was initiated. Chemotherapy was initiated with rituximab and cyclophosphamide with dexamethasone. An attempt to transfer the patient to a tertiary care academic center for continued aggressive management was initiated, however, declined due to insurance and capacity reasons. The patient eventually left the facility against medical advice.

## Discussion

Burkitt lymphoma (BL) is a highly aggressive type of non-Hodgkin B-cell lymphoma, which accounts for 1-2% of all cases of non-Hodgkin lymphoma (NHL) in the general population [2]. BL was initially described in 1958 as a sarcoma of the jaw that predominantly affected children [3]. Since then, three clinical variants of BL have been described: endemic, sporadic, and immunodeficiency-related (e.g., associated with HIV). The endemic form occurs predominantly in Africa and the Middle East in children aged 4-7 with a 2:1 predilection for males and involves bones of the jaw and other facial bones in 50% of cases, as well as mesenteric and central nervous system (CNS) spread [4]. Nearly 100% of these patients are infected by Epstein-Barr Virus (EBV); the immunodeficiency-associated form occurs primarily in HIV-infected patients and accounts for 30-40% of NHL in this population. These cases tend to occur at younger ages and higher median CD4 counts (>200) than the diffuse B-cell type, often presenting as the first AIDS-defining criterion. The sporadic form occurs worldwide and represents most cases occurring in the United States and Europe with a predilection for males. The clinical presentation of BL is variable, however, the CNS, bone marrow, and gastrointestinal tract, particularly the ileocecal area, are the most frequently involved sites [1]. HIV-associated BL has more extranodal disease. The first manifestation of HIV-associated BL is often related to the site of extranodal involvement, and gastrointestinal tract and bone marrow are the most common [1].

Burkitt’s lymphoma occurs secondary to the overexpression of the MYC oncogene located on the long arm of chromosome 8 (8q24). The most common translocation is between the c-MYC proto-oncogene and the IgH (Immunoglobulin-H) genes and accounts for 80% of cases [5]. The mature intermediate-sized B-cells of BL have a very high proliferative rate approaching 100% with numerous admixed tangible body macrophages. The high proliferative index leads to spontaneous apoptosis of tumor cells which are ingested by macrophages giving this disease the pathognomonic “starry-sky appearance” morphologically [4]. The growth fraction as measured by Ki67 staining approaches 100%. BL is derived from germinal center B-cells, and these cells are positive for CD10, CD19, CD20, CD79a, CD45, and BCL6 and negative for terminal deoxynucleotidyl transferase (TdT) as well as BCL2. BL is highly sensitive to treatment with intensive combination cytotoxic chemotherapy, with caution exercised to prevent tumor lysis syndrome [6].

Sporadic BL accounts for less than 1% of adult NHLs in the United States [1]. Sporadic BL is more common among Caucasians than African or Asian Americans [7]. The abdomen, particularly the ileocecal area, is the most frequent site of origin. Mesenteric and retroperitoneal lymph nodes are commonly involved. Extranodal involvement in sporadic BL may occur in the stomach, duodenum, distal ileum, cecum mesentery, liver, spleen, pancreas, kidney, testis, ovaries, and CNS [1]. In adults, sporadic BL typically presents with diffuse abdominal involvement and localized lymphadenopathy. Symptoms include abdominal pain, nausea, vomiting, bowel obstruction, gastrointestinal bleeding, and distension secondary to ascites. Extranodal disease in sporadic BL presents in 23%-85% of all cases, 50% of which occur in the gastrointestinal tract. Central nervous system (CNS) and bone marrow (BM) involvement account for 13-17% and 13%-40% of all BL respectively. Abdominal tumors in the terminal ileum with ileal or ileocolic intussusception caused by BL, as a cause of acute abdomen-mimicking appendicitis, have been reported [8]. The rapid development of peritoneal lymphomatosis is another rare presentation of extranodal lymphoma, usually associated with diffuse B cell lymphoma, and also seen in sporadic BL [9,10]. Involvement of the BM and the CNS is indicative of progressive disease and is reported in 13-17% of adults [11,12].

Patients with sporadic BL usually present with advanced-stage and bulky abdominal disease caused by a short doubling time of the tumor [1]. One case report in the literature talks about a 67-year-old patient with a similar clinical presentation as our patient, in which colonoscopy proved beneficial. In this patient, however, CT of the abdomen showed peritoneal carcinomatosis present in a small amount of ascitic fluid, and a small lesion in the hepatic flexure [13]. Hence despite the absence of a significant abdominal tumor burden, the CT findings showing colonic involvement led to the prompt diagnosis by colonoscopy with biopsy. In comparison, our patient did not present with bulky disease, obscuring the diagnosis, to begin with. The absence of any CT findings to point us towards this malignancy was also absent. Our case demonstrates a peculiar presentation of Burkitt’s lymphoma with extra-nodal involvement, hepatosplenomegaly, and biopsy positive-cecum polyps. Additionally, our patient's age of presentation is atypical given that the age of presentation is most common in the elderly.

The clinical presentation of BL is non-specific, which makes it diagnostically challenging. Histologic diagnosis is essential to distinguish BL from other aggressive B-cell lymphomas. Laboratory tests should include routine hematology and biochemistry panels including lactate dehydrogenase (LDH), uric acid, calcium, and phosphorus as most patients are at high risk for tumor lysis syndrome. HIV and hepatitis B and C testing should be performed routinely. A bone marrow biopsy is essential. Cerebrospinal fluid (CSF) analysis by cytology and flow cytometry should be performed if there is CNS involvement. CT or PET is commonly used for tumor staging [6]. Ultimately, because of the rarity of the disease, our case highlights the importance of maintaining BL as a differential diagnosis.

## Conclusions

The presentation of Burkitt lymphoma is highly variable and thus represents a diagnostic challenge. It is crucial to make a histologic diagnosis to distinguish BL from other aggressive B-cell lymphomas. This case exemplifies a unique presentation of an investigative journey to the diagnosis of BL via biopsy of a cecal polyp in the absence of an intra-abdominal tumor. Our case report elucidates the notion that colonoscopy can be an important tool in the histological confirmation of this disease.
